# Reclassification of the *BRAF* p.Ile208Val variant by case-level data sharing

**DOI:** 10.1101/mcs.a002675

**Published:** 2018-10

**Authors:** Andrew R. Grant, Sarah E. Hemphill, Lisa M. Vincent, Heidi L. Rehm

**Affiliations:** 1 Laboratory for Molecular Medicine, Partners Healthcare Personalized Medicine, Cambridge, Massachusetts 02139, USA;; 2 GeneDx, Gaithersburg, Maryland 20877, USA;; 3 Center for Genomic Medicine, Massachusetts General Hospital, Boston, Massachusetts 02114, USA;; 4 The Broad Institute of Harvard and MIT, Cambridge, Massachusetts 02142, USA

**Keywords:** increased nuchal translucency, intellectual disability, moderate, short stature, webbed neck

## Abstract

The ClinVar database is a useful tool for patients and physicians to view variant interpretations submitted by clinical and nonclinical labs. However, variants of uncertain significance (VUS) in ClinVar can pose a significant burden on patients. If possible, it is important to resolve discrepancies and uncertainties surrounding interpreted variants. Here we highlight a case of a family who received a report of a variant (c.622A>G, p.Ile208Val) in *BRAF* following prenatal RASopathy testing. The variant had been previously classified by our laboratory as a VUS, so the mother contacted our laboratory via ClinVar for further information, which prompted reevaluation of the variant. Multiple sources of case-level data as well as the presence of the variant in the general population yielded sufficient evidence to reclassify the variant as likely benign. This reclassification alleviated significant concern for the family, and the child was born healthy with no clinical manifestations of Noonan syndrome or a RASopathy.

## CASE PRESENTATIONS

Our laboratory was contacted by the mother of Proband 1. She had undergone prenatal genetic testing for Noonan syndrome following a nuchal translucency (NT) measurement of 3.2 mm. Although amniocentesis and ultrasound at 20 wk were both normal, increased NT measurements have been shown to be associated with an increased risk of developing Noonan syndrome (Nisbet et al. 1999). There was no family history reported of clinical features of Noonan or Noonan-related syndromes. The fetus was found to have a missense variant, c.622A>G (p.Ile208Val), in *BRAF*. Somatic variants in *BRAF* are well-documented in various cancers (Holderfield et al. 2014; Halaban and Krauthammer 2016) and germline variants have been reported in individuals with RASopathies. Of note, at least nine *BRAF* missense variants have been observed in patients with Noonan syndrome, five of them de novo occurrences (Razzaque et al. 2007; Nystrom et al. 2008; Sarkozy et al. 2009; Lee et al. 2011; van Trier et al. 2016). At that time, the p.Ile208Val variant was reported to the patient as a variant of uncertain significance (VUS), which was consistent with interpretations in ClinVar (Variation ID 174180), by GeneDx and the Partners Laboratory for Molecular Medicine (LMM). Given the abnormal NT result and the uncertainty surrounding the clinical significance of this variant, the patient was highly concerned about the outcome of the pregnancy and sought further information about the variant. To determine the origin of the variant in Proband 1, both the mother and the father were screened for the variant and the father was found to be heterozygous. The father was healthy and had no clinical features of a RASopathy.

Previously, the p.Ile208Val variant was identified at our laboratory in an another individual (Proband 2) with middle aortic syndrome, low nasal bridge, hypertension, facial coarseness, short stature, learning disabilities/mental retardation, wide-spaced nipples, and webbed neck and a reportedly unaffected parent. Proband 2 underwent a next-generation sequencing panel of 12 genes associated with RASopathies (*PTPN11*, *SOS1*, *RAF1*, *KRAS*, *NRAS*, *BRAF*, *MEK1*, *MEK2*, *HRAS*, *SHOC2* (exon 02), *CBL*, and *SPRED1*) and no other variants in any of these genes were identified.

This variant was also observed at GeneDx (ClinVar submission SCV000329576.4), first in 2016. Upon reassessment, GeneDx noted this variant was observed in three unrelated families undergoing exome testing. The clinical presentations of individuals with the variant (*n* = 6) were either normal or had clinical features inconsistent with a RASopathy.

## VARIANT INTERPRETATION

The p.Ile208Val variant in *BRAF* was classified as uncertain significance by the LMM in April 2013 (Duzkale et al. 2013) when it was detected in Proband 2 and an unaffected parent, described above. It was absent from the Exome Sequencing Project database (http://evs.gs.washington.edu/EVS/) and computational tools (SIFT, PolyPhen-2, and AlignGVGD) and conservation analysis did not provide strong support for or against the variant's impact on protein function. The variant had been reported as a somatic mutation in a melanoma cell line. However, the cell line also carried the p.Val600Glu variant in *BRAF*, a well-characterized activating mutation (Ikediobi et al. 2006). Nevertheless, the presence of a variant in a cancerous cell line does not provide evidence for pathogenicity of the variant in a germline state. Therefore, with the information available at the time, the clinical significance of the p.Ile208Val variant was uncertain.

Our laboratory reassessed the p.Ile208Val variant in July 2017 upon being contacted by the mother of Proband 1. At that time, the variant had been identified in 3/126430 European alleles in the Genome Aggregation Database (gnomAD; gnomad.broadinstitute.org). It is important to note that although Noonan syndrome is highly penetrant, it has also been found to have highly variable expressivity (Zenker et al. 2004). Therefore, the presence of the variant in three individuals in the general population whose phenotypes are unavailable would not be sufficient evidence alone to rule out pathogenicity. However, we applied the ACMG/AMP BS2 rule per the RASopathy Expert Panel's specifications which asserts that the presence of a RASopathy variant in multiple healthy, phenotyped adult individuals is strong evidence that the variant is benign (Richards et al. 2015; Gelb et al. 2018). The p.Ile208Val variant has been observed in at least three phenotyped individuals without evidence of a RASopathy at the LMM and GeneDx. The BS1 threshold for RASopathy variants is 0.025% (Gelb et al. 2018), which is used as a strong piece of evidence toward benign when the frequency of the variant in the general population (i.e., gnomAD) is higher than this threshold. The RASopathy Expert Panel currently does not specify the use of less conservative allele frequency thresholds for strength lowering modifications to BS1. However, using expert judgment, the presence of this variant in three individuals in gnomAD and three tested families from GeneDx without clinical features of a RASopathy was deemed worthy of applying BS1 at a supporting, rather than strong level of strength. These criteria were sufficient to classify the variant as likely benign. An updated interpretation was submitted to ClinVar by the LMM in November 2017 (SCV000205449.4). GeneDx also reclassified the variant as likely benign (SCV000329576.6).

## SUMMARY

In this example, personal communication from a patient led to reclassification of the p.Ile208Val variant in *BRAF* from VUS to likely benign by two clinical testing laboratories. Proband 1, presented here, was a fetus who was later born healthy with no features of a RASopathy. Proband 2 had some clinical features of a RASopathy, but inherited the variant from an unaffected parent. Finally, the variant was observed in six individuals from three families without RASopathy indications who underwent whole-exome sequencing at GeneDx. Therefore, the reclassification is supported by clinical observations of the variant in multiple individuals without phenotypes consistent with a RASopathy.

The ability for the patient to contact our laboratory through ClinVar was integral to the reclassification of this variant. Furthermore, this variant is rare, present in only 0.002% of alleles in the European population, and it is likely that it would not have been seen by our laboratory again. Therefore, the variant may not have been reevaluated and reclassified without patient engagement. Although efforts to resolve discrepancies in classifications by clinical laboratories via standardized interpretation guidelines and data sharing have been successful (Garber et al. 2016; Harrison et al. 2017), concordant VUS interpretations would not be included in such studies and may never be otherwise prompted for reassessment.

The value of genomic data sharing is widely recognized and ClinVar is a valuable tool to facilitate sharing. Although patient privacy and consent is a consideration, a recent article concluded that explicit individual consent is not required for sharing de-identified variant information to ClinVar and including relevant phenotypic information is acceptable (Azzariti et al. 2017). We therefore encourage laboratories to include structured phenotype data in their submissions to ClinVar to aid in aggregating case-level evidence.

The original VUS result posed a significant emotional burden on the family of Proband 1 which was mitigated by this reclassification. This example demonstrates that in some cases, patients themselves can prompt variant reclassification by sharing their own data. And whereas we encourage use of the ClinVar database by patients, it is worth contemplating a sustainable model for patient engagement given the constant knowledge evolution of genomic variation. We propose that patients should be encouraged to track their variants in ClinVar so they may be alerted to changes in classification; however, we suggest that the best model for follow-up when changes are seen is for patients to contact their physician and have the physician engage the laboratories on their behalf.

We also suggest patients submit genomic testing results to the GenomeConnect registry (www.genomeconnect.org) to participate in ClinGen's data sharing efforts, which include being updated when a classification in ClinVar, from the reporting laboratory, changes.

## ADDITIONAL INFORMATION

### Data Deposition and Access

The Partners LMM variant was submitted to ClinVar (http://www.ncbi.nlm.nih.gov/clinvar/) and can be found under accession number SCV000205449.5; the GeneDx submission can be found under accession number SCV000329576.7.

### Ethics Statement

This study was conducted under IRB protocols approved by the Partners Human Research Committee Protocol Number 2013P001099 (LMM) and the Western Institutional Review Board, Study Number 1169768, WIRB Pro Number 20162523 (GeneDx) that state that this research meets the requirements for a waiver of additional consent.

### Acknowledgments

We thank the family for their generosity in sharing clinical data and laboratories for their ongoing data sharing in ClinVar.

### Author Contributions

A.R.G. and S.E.H. wrote the manuscript. H.L.R. communicated with the family (Proband 1). H.L.R. and L.M.V. reclassified the variant on behalf of the LMM and GeneDx, respectively, and edited the manuscript.

### Funding

This study was supported in part by the National Human Genome Research Institute (NHGRI) in conjunction with funding from the Eunice Kennedy Shriver National Institute of Child Health and Human Development (NICHD) under award U41HG006834. The content is solely the responsibility of the authors and does not necessarily represent the official views of the National Institutes of Health.

### Competing Interest Statement

The authors have declared no competing interest.
